# Opportunities arising from the COVID-19: an international orthopaedic surgeons’ perspective

**DOI:** 10.1007/s00590-022-03334-8

**Published:** 2022-09-02

**Authors:** Anthony Howard, Tom Robinson, Amy Lind, Sophanit Pepple, COVID Ortho Group Collaborators, George D. Chloros, Peter V. Giannoudis

**Affiliations:** 1 Academic Department of Trauma and Orthopaedics, School of Medicine, Leeds Teaching Hospital, University of Leeds, George Street, Leeds, LS9 7TF UK; 2 Nuffield Department of Orthopaedics, Rheumatology and Musculoskeletal Sciences, Oxford, UK; 3 NIHR Leeds Biomedical Research Center, Chapel Allerton Hospital, Leeds, UK

**Keywords:** COVID-19, Lessons, Positive, Change, Orthopaedic surgery

## Abstract

**Purpose:**

The unprecedented COVID-19 experience has posed severe challenges to the health care system and several of these are documented in orthopaedic surgery; however, although the pandemic has also brought positive changes, these have not been precisely documented. The purpose of this survey is to identify positive perceptions by orthopaedic surgeons at an international level.

**Methods:**

A cross-sectional, web-based survey inviting 120 orthopaedic surgeons was conducted in April 2020 querying about the positive lessons COVID-19 would teach us. From all responses, thematic codes were obtained and an exploratory thematic analysis was carried out to determine the prevalent themes.

**Results:**

A total of 100 responses (83% response rate) from a total of seven countries were received. The variety of responses received were grouped into 13 different thematic codes. The thematic analysis generated two major themes: “Virtual reorganization” and “Wellness and sustainability”. Fifty-four per cent of the participants reported positive changes in service reorganization and virtual consultation, whereas 30% replied with an increased feeling of well-being which overlapped with environmental benefits, including reduced paperwork, reduced travelling and increased quality time for family and reflection.

**Conclusions:**

Despite the negative aspects of the pandemic, responders reported several positive changes particularly relating to service reorganization and personal well-being. This study prompts further larger scale research to unravel further detail in those positive aspects and strongly enhance our future orthopaedic practice.

## Introduction

Due to the COVID-19 pandemic, there have been unpreceded strain on health care systems [1], which abruptly forced them to differently manage patients in the face of this potentially overwhelming demand [2, 3]. There have been several negative aspects of the pandemic affecting orthopaedic surgery, including shutdown of planned operating with raised thresholds for the operative management of injuries and the re-deployment of staff to support colleagues in overwhelmed specialties [4–11]. This has resulted in massive waiting lists for elective surgery, reduced training opportunities and increased negative mental health effects in staff members [6, 12, 13].

Adversity is also an opportunity for innovation [14], and undoubtedly, there has also been a positive shift in response to the challenges, including the considerable increased use of digitized resources in the form of virtual consultations, meetings, educational tools [15, 16], as well as an enhanced international research collaboration [17, 18]. Some of those positive aspects have been reported in populations encompassing general specialties, [19, 20] but not specifically for orthopaedics.

Although the negative effects of the pandemic have been previously reported in several publications pertaining to orthopaedic surgeons [5–9], the aforementioned positive changes brought to our orthopaedic practice by the pandemic have not been precisely recorded. Therefore, the rationale for this study is to conduct a global survey to record those positive experiences during the pandemic specifically for orthopaedic surgery, and thus capture the new ideas, perceptions and lessons learned which in turn can be used to promote future innovation and improvement in our everyday practice of orthopaedics.

## Methods

During April 2020, 120 international orthopaedic surgeons registered on the collaboration list of our Institution were sent an electronic invitation to participate in a cross-sectional, web-based survey which was kept live until April 2021. To reduce the chance on bias and encourage a wide response, a single question was devised, “What are the positives lessons covid could teach us” with no restriction on the text the participant can write. Ethical approval was given by the School of Medicine Ethics Committee, Leeds University, MREC 20-XXX.

An exploratory thematic analysis was employed based on the six-phase approach outlined by Braun and Clarke [21]. This methodology was selected because of the nature of the dialogue and its flexibility, comparative to other techniques, for example, discourse analysis. Two of the authors reviewed all the participants responses and formulated codes after familiarization and review of data. All responses were coded using the direct quotes from the participants’ responses using NVIVO V1.5 software (NVivo qualitative data analysis Software; QSR International Pty Ltd.). The codes generated were also correlated to the participants country.

Exploratory thematic analysis was performed until saturation was achieved with no new codes or themes being generated [22]. The themes were reviewed by the research team, named and finalized. Disagreements in coding or theme production were discussed within the team until a consensus could be reached.

## Results

**Fig. 1:**
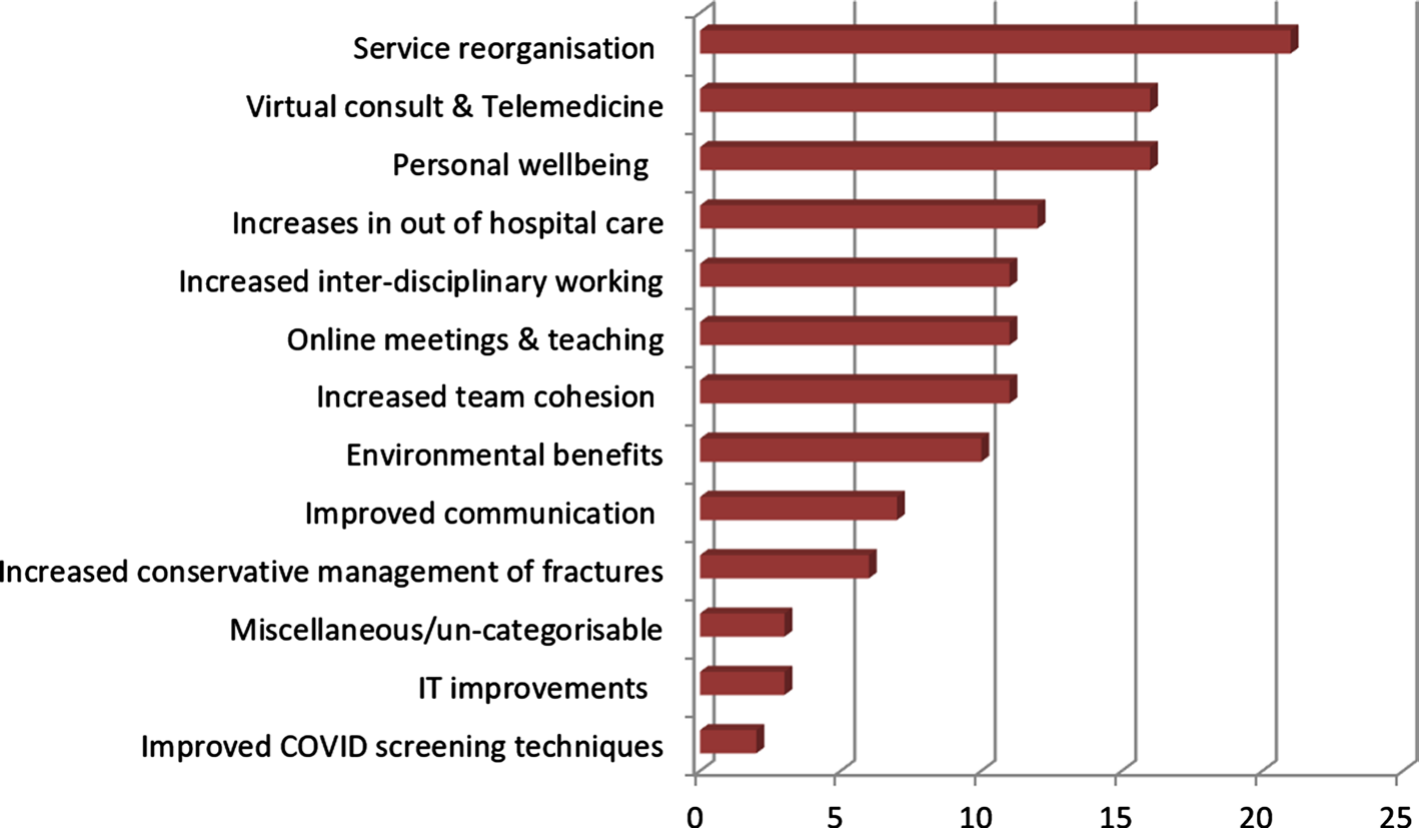
The total number of codes generated, in descending rank order

**Fig. 2:**
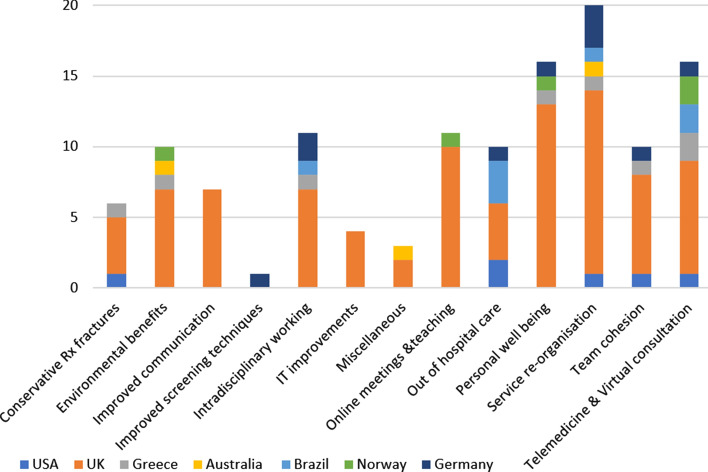
Distributions of codes by participant country

A total of 100 responses were received via the website (83% response rate), with responders being based in Australia, Brazil, Germany, Greece, UK and the USA. Based on 129 response-types, the 13 codes generated by the consensus as outlined above are shown in descending order. (Fig. 1) Responses showing the country distribution per code are shown. (Fig. 2).

The thematic analysis generated two major themes:

### Theme 1—virtual reorganization

It was found 54% of participants whose submission reported positive changes to the way their departments services had been organized (code: “Service reorganization) also reported positive use of virtual consultations/telemedicine (code: “Virtual consult & Telemedicine”). Typical response examples are shown:

*Example 1*: “Remote telephone fracture clinics were implemented wherever possible, especially for follow-up appointments, and were largely successful. This in itself has changed the way we will practice when the pandemic is finally over.”

Within this theme, there was a high degree of support of a new way or working that improved the efficiency of the service provision and definitive clinical management.

*Example 2*: “All patients with hand and wrist injuries were initially evaluated by a team of 3 fellows (that were on-call with daily rotation) and then photos and videos of trauma and radiographs were sent to a senior surgeon who gave detailed instructions for conservative or surgical treatment at the outpatient department”.

### Theme 2, wellness and sustainability

**Fig. 3:**
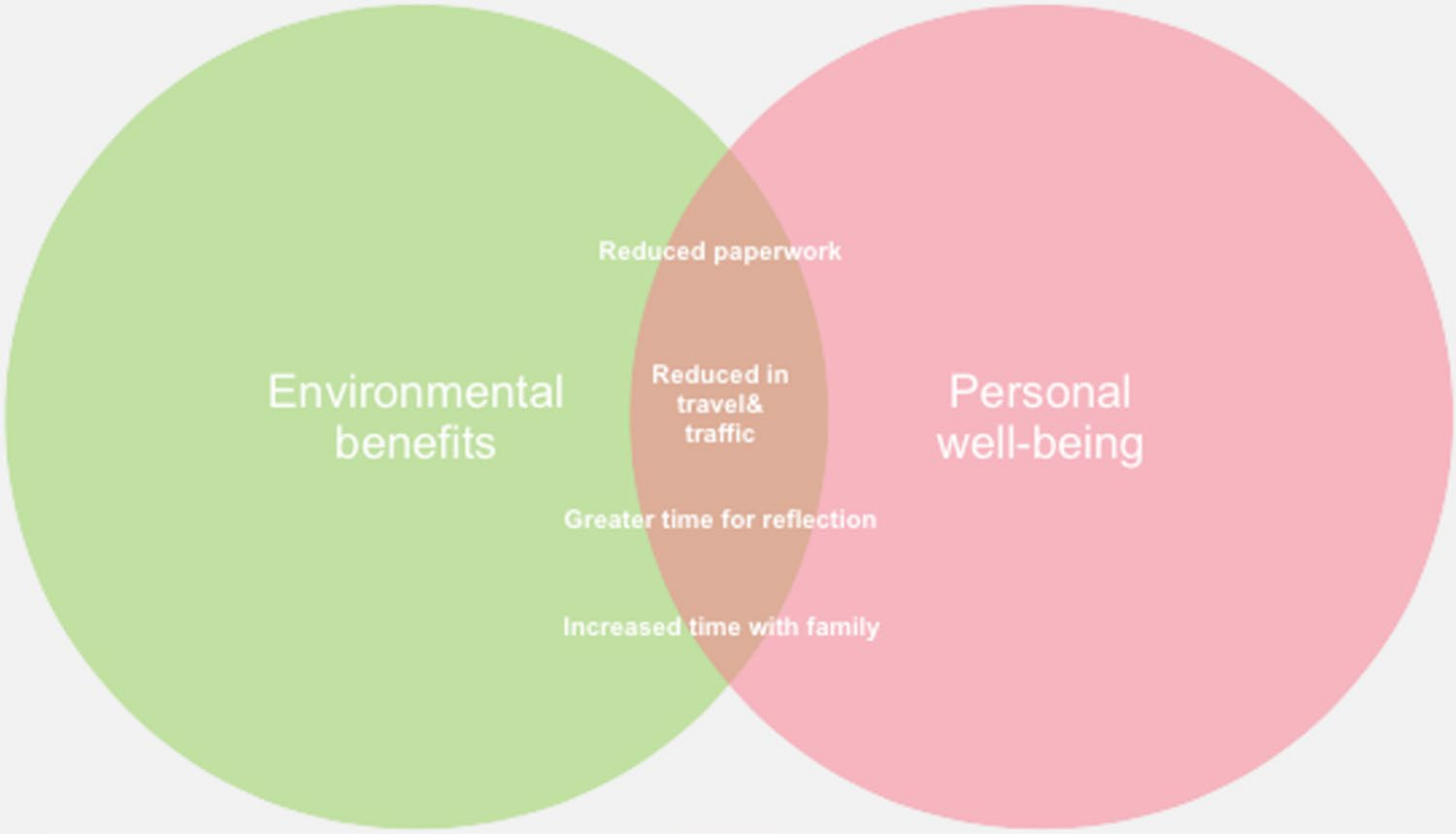
Theme 2: Wellness and sustainability. 62% of responders whose submission coded for an increased feeling of personal well-being were also coded for changes to working practices that benefited the environment. Figure also displays quotes from these submissions

Within the Theme 2, 30% of all participants responded with statements that coded for an increased feeling of well-being (code: “Personal wellbeing”). This was the third most common code used in the study. Participants who reported an increased feeling of personal well-being were more likely to also state an environmental benefit (code: “Environmental Benefits”) from the ways of working that had been adopted due to the pandemic. There was overlap between environment and personal well-being, with reduced paperwork, reduced travelling, and great time for family and reflection (Fig. 3). Direct quotes that related to this theme are shown as follows:

*Example 3*: “Life during the pandemic has been [..] quiet, no traffic, no air pollution and family reunion”.

*Example 4*: “No long commute means more energy and enthusiasm”.

*Example 5*: “We are gaining knowledge not only from within the region but national [sic] without contributing to carbon footprint”.

## Discussion

There is no doubt that the pandemic resulted in an unprecedented challenge to the health care services including putting significant pressure on staff of all specialties [1]. However, the vast majority of publications on orthopaedic surgeons has focussed on the intuitive negative aspects [5–11]; nevertheless, any experience may also have a positive impact. The aim of this study was to collate the positive experiences reported by orthopaedic surgeons to identify important lessons for our future practice.

The main results of the study via the thematic analysis of the 100 responses received are that the two main themes that emerged were “Virtual reorganization” and “Wellness and Sustainability”. As far as the first one, it seems that the pandemic has a positive influence in making the system better. As outlined by the responder in example 1, it is likely that at least some aspects of the virtual model used during the pandemic will continue into future practice. Previously established virtual clinic models that were used pre-pandemic have shown good outcomes for cost-effectiveness and patient satisfaction [23, 24]. Early indicators from the pandemic experience have shown similarly positive outcomes [25]. In addition to the changes to clinical practice, the move towards virtual meetings for educational and administrative purposes have been well-accepted [26–28]. This has also been demonstrated in a recent study of 131 National Health System (NHS) staff members of various specialties, showed that 56% felt that the NHS would benefit from a positive change as a result of the pandemic and that this would be long lasting [19]. Another study examining the pandemic effects on orthopaedic surgeons in British Columbia mostly concluded negative aspects of the pandemic; however, 91% of respondents reported that they would definitely implement telemedicine in the long term, which was scare before the pandemic [10].

Regarding the finding that almost a third of respondents experienced an increased sense of personal well-being was definitely surprising. The negative effects on the mental health of health care staff working during the pandemic are well documented [29]. In addition, prior to the pandemic, globally high levels of burnout amongst orthopaedic surgeons had been identified [30–32], and recent studies are showing that this increased in the pandemic [6]. However, a recent Dutch study focussing on physician’s positive work experience during the pandemic showed that physicians perceived employability was significantly higher during than prior to the pandemic, the workload was less and this together with the high appreciation by society are likely to contribute to the increased sense of well-being [20]. During the pandemic, the reduced level of social and travel activity has undoubtedly enabled individuals to reflect and appreciate the increased time on their hands. Institutions have seen a reduction in contact hours and travel time compared to pre-pandemic levels, as echoed by statements in examples 3 and 4. Better sleeping patterns and reduced hours contribute to a reduction in the perception of work overload [33]. Finally, the impending fear of health care being overwhelmed, combined with greater teamwork counter intuitively seems to have generated a perceived better work environment. Within most areas of medicine, there are staff shortages and an urgent need to recruit into the profession [34]. It is imperative that the lesson learnt in terms of the positive work environment is carried forward to help to address the deficient number of medical personnel.

There are several limitations to the study: Firstly, the sample size was relatively small. We received 100 responses from seven countries who closely collaborate with our Institution, which is a small sample size relative to the worldwide orthopaedic surgical population. Nonetheless, the purpose of this study was to document and promote the reported positive experiences from the pandemic in order that the opportunity for learning from adversity amongst the orthopaedic community is not lost. Second, there are more sophisticated methods that could have been employed such as a Delphi analysis, but our study shows an important insight which the authors hope is useful during the current phase of returning towards more normal health care provision. Finally, the responders to surveys such as this study are self-selecting in their choice to response, which leads to inevitable bias.

## Conclusion

The majority of studies on orthopaedic surgeons’ perceptions on COVID-19 report negative experiences. However, the literature focussing on all specialties has shown that this pandemic may also bring out positive aspects. In this survey, orthopaedic surgeons have reported that there have positive changes in the workplace, related to virtual reorganization, as well as improvements in their sense of well-being that is likely in part attributable to a reduction in working hours and time spent commuting. In particular, the use of telemedicine and virtual consultation have changed dramatically the way we practice and unexpectedly, staff well-being has increased. Further larger scale cohort research that would unravel more detail about the positive aspects and lessons learnt during the pandemic would be extremely useful in order to disseminate the positive experiences and further enhance our future orthopaedic practices.

## References

[1] Blumenthal D, Fowler EJ, Abrams M, Collins SR (2020). Covid-19—implications for the health care system. N Engl J Med.

[2] Hale T, Angrist N, Goldszmidt R, Kira B, Petherick A, Phillips T (2021). A global panel database of pandemic policies (Oxford COVID-19 government response tracker). Nat Hum Behav.

[3] Unruh L, Allin S, Marchildon G, Burke S, Barry S, Siersbaek R (2021). A comparison of 2020 health policy responses to the COVID-19 pandemic in Canada, Ireland, the United Kingdom and the United States of America. Health Policy.

[4] Wallace CN, Kontoghiorghe C, Kayani B, Chang JS, Haddad FS (2020). The impact of COVID-19 on trauma and orthopaedic surgery in the United Kingdom. Bone Joint Open.

[5] Chatterji G, Patel Y, Jain V, Geevarughese NM, Haq RU (2021). Impact of COVID-19 on orthopaedic care and practice: a rapid review. Indian J Orthop.

[6] Kołodziej Ł, Ciechanowicz D, Rola H, Wołyński S, Wawrzyniak H, Rydzewska K (2021). The impact of the COVID-19 pandemic on polish orthopedics, in particular on the level of stress among orthopedic surgeons and the education process. PLoS ONE.

[7] Meraghni N, Bouyoucef H, Larbi R, Soal N, Benkaidali R, Derradji M (2021). Orthopaedic surgeons´ perceptions and attitudes on COVID-19 related changes in practice: an international cross-sectional survey. Pan Afr Med J.

[8] Muzzammil M, Jahanzeb S, Asghar A, Jabbar S, Waheed H (2021). Impact of the COVID-19 pandemic on orthopedic surgeon in Pakistan. Int J Res Orthop.

[9] Ranuccio F, Tarducci L, Familiari F, Mastroianni V, Giuzio E (2020). Disruptive effect of COVID-19 on orthopaedic daily practice: a cross-sectional survey. JBJS.

[10] Simon MJK, Regan WD (2021). COVID-19 pandemic effects on orthopaedic surgeons in British Columbia. J Orthop Surg Res.

[11] Teo SH, Abd Rahim MR, Nizlan NM (2020). The impact of COVID-19 pandemic on orthopaedic specialty in Malaysia: a cross-sectional survey. J Orthop Surg.

[12] Thakrar A, Raheem A, Chui K, Karam E, Wickramarachchi L, Chin K (2020). Trauma and orthopaedic team members’ mental health during the COVID-19 pandemic. Bone Joint Open.

[13] Wong JSH, Cheung KMC (2020). Impact of COVID-19 on Orthopaedic and trauma service: an epidemiological study. J Bone Joint Surg.

[14] Howard A, Kanakaris NK, Giannoudis PV (2020). Turning adversity and deprivation into improvements in medicine—the COVID opportunity. Injury.

[15] Woolliscroft JO (2020). Innovation in response to the COVID-19 pandemic crisis. Acad Med.

[16] Powis S, Philip P (2020) Clinical innovations during COVID-19. Board meetings in common of the boards of NHS England and NHS improvement, 28 July 2020

[17] Aviv-Reuven S, Rosenfeld A (2021). Publication patterns' changes due to the COVID-19 pandemic: a longitudinal and short-term scientometric analysis. Scientometrics.

[18] Fry CV, Cai X, Zhang Y, Wagner CS (2020). Consolidation in a crisis: patterns of international collaboration in early COVID-19 research. PLoS ONE.

[19] Barr-Keenan R, Fay T, Radulovic A, Shetty S (2021). Identifying positive change within the NHS as a result of the COVID-19 pandemic. Future Healthc J.

[20] van Leeuwen EH, Taris T, van Rensen ELJ, Knies E, Lammers J-W (2021). Positive impact of the COVID-19 pandemic? A longitudinal study on the impact of the COVID-19 pandemic on physicians’ work experiences and employability. BMJ Open.

[21] Braun V, Clarke V (2006). Using thematic analysis in psychology. Qual Res Psychol.

[22] Damayanthi S (2019). Thematic analysis of interview data in the context of management controls research.

[23] Khan SA, Asokan A, Handford C, Logan P, Moores T (2020). How useful are virtual fracture clinics?: a systematic review. Bone Joint Open.

[24] Reilly MO, Breathnach O, Conlon B, Kiernan C, Sheehan E (2019). Trauma assessment clinic: virtually a safe and smarter way of managing trauma care in Ireland. Injury.

[25] Murphy EP, Fenelon C, Murphy RP, O'Sullivan MD, Pomeroy E, Sheehan E (2020). Are virtual fracture clinics during the COVID-19 pandemic a potential alternative for delivering fracture care? A systematic review. Clin Orthop Relat Res.

[26] Burchette D, To C, Willmott H (2021). Introduction of a virtual trauma meeting in response to COVID-19. Ann Royal Coll Surg Engl.

[27] Essilfie AA, Hurley ET, Strauss EJ, Alaia MJ (2020). Resident, fellow, and attending perception of E-learning during the COVID-19 pandemic and implications on future orthopaedic education. J Am Acad Orthop Surg.

[28] Rajasekaran RB, Whitwell D, Cosker TDA, Gibbons CLMH, Carr A (2021). Will virtual multidisciplinary team meetings become the norm for musculoskeletal oncology care following the COVID-19 pandemic? - experience from a tertiary sarcoma centre. BMC Musculoskelet Disord.

[29] De Kock JH, Latham HA, Leslie SJ, Grindle M, Munoz S-A, Ellis L (2021). A rapid review of the impact of COVID-19 on the mental health of healthcare workers: implications for supporting psychological well-being. BMC Public Health.

[30] Arora M, Diwan AD, Harris IA (2013). Burnout in orthopaedic surgeons: a review. ANZ J Surg.

[31] Faivre G, Marillier G, Nallet J, Nezelof S, Clment I, Obert L (2019). Are French orthopedic and trauma surgeons affected by burnout? Results of a nationwide survey. Orthop Traumatol Surg Res.

[32] Siddiqui AA, Jamil M, Kaimkhani GM, Wasim M, Katto MS, Yaqoob U (2018). Burnout among orthopedic surgeons and residents in Pakistan. Cureus..

[33] Firth-Cozens J (2001). Interventions to improve physicians’ well-being and patient care. Soc Sci Med.

[34] Haleem A, Javaid M, Vaishya R, Vaish A (2020). Effects of COVID-19 pandemic in the field of orthopaedics. J Clin Orthop Trauma.

